# Structural characteristics of locust bean gum hydrolysate and its alleviating effect on dextran sulfate sodium-induced colitis

**DOI:** 10.3389/fmicb.2022.985725

**Published:** 2022-08-10

**Authors:** Kangjia Jiang, Duo Wang, Le Su, Xinli Liu, Qiulin Yue, Baojun Li, Kunlun Li, Song Zhang, Lin Zhao

**Affiliations:** ^1^State Key Laboratory of Bio-Based Material and Green Papermaking, School of Bioengineering, Qilu University of Technology, Shandong Academy of Sciences, Jinan, China; ^2^Shandong Zhuoran Biotechnology Co., Ltd., Jinan, China; ^3^Shandong Chenzhang Biotechnology Co., Ltd., Jinan, China

**Keywords:** ulcerative colitis, locust bean gum hydrolysate, anti-inflammatory effect, NF-κB, gut microbiota

## Abstract

**Background:**

Ulcerative colitis (UC) is an inflammatory lesion of the colon from various causes. As current therapeutic drugs have adverse effects on patients with UC, there is a growing demand for alternative medicines from natural and functional foods. Locust bean gum, as a dietary fiber, has a variety of physiological effects.

**Methods:**

In the present study, locust bean gum hydrolysate (LBGH) was obtained from the acid hydrolysis of locust bean gum. The structure of LBGH was characterized by thin-layer chromatography and high performance liquid chromatography (HPLC)-electrospray ionization (ESI)-mass spectrometry (MS)/MS analysis. And we investigated the therapeutic effect of LBGH on a mouse model of dextran sulfate sodium (DSS)-induced colitis.

**Results:**

It was observed that the LBGH consisted of a mixture of monosaccharides and oligosaccharides with a degree of polymerization (DP) 2–7. LBGH treatment dramatically alleviated colonic pathological damage, suppressed the overproduction of pro-inflammatory factors and the activation of nuclear factor κB (NF-κB), increased the mRNA expression of tight junction proteins, and increased the abundance of probiotics such as *Lactobacillus* and *Bifidobacterium* in the gut.

**Conclusion:**

There is a correlation between these mitigating effects on inflammation and the treatment of LBGH. Therefore, LBGH has tremendous potential in the treatment of colitis.

## Introduction

Ulcerative colitis (UC) is a chronic autoimmune disease with inflammation mainly in the mucosa and submucosa of the colon (Yamamoto-Furusho et al., 2020). In the past decade, over 1–5 million people in North America and 2 million in Europe suffered from this disease. However, in recent years, the incidence and prevalence patterns of UC have changed worldwide. An increasing trend in the incidence of UC has been reported in Asia (Wei et al., 2021). The incidence of colitis in Asia and the Middle East has been reported to be 0.15–6.5 per 100,000 person-years in recent years (Sharara et al., 2018). The survey proved that the recurrence rate of UC patients is 57.69% (Azad et al., 2011; Kaplan, 2015; Ng et al., 2017). Therefore, it is urgent to find some natural products as new and safer therapeutic drugs.

Up to now, the pathogenesis of UC is still not quite clear. Studies confirmed that NF-κB is a major inflammatory signaling pathway (Chen et al., 2017). Activation of NF-κB induces the production of inflammatory cytokines, such as TNF-α, IL-1β, and IL-6 (Amirshahrokhi, 2019), which increase the inflammatory response.

It is increasingly believed that environmental factors and intestinal microbiota also have an important impact on the occurrence and development of UC (Jairath and Feagan, 2020; Kobayashi et al., 2020). Peng et al. (2013) suggested that the persistent inflammatory mechanisms of UC might be the result of an interaction between the gut microbiota, inflammatory signaling, and tissue remodeling (Tang et al., 2021). Altered microbial composition and function in UC lead to increased immune stimulation, epithelial dysfunction, or increased mucosal permeability (Sartor, 2008). Gut microbial community with lower diversity and stability is observed in UC patients. In particular, *Bacteroides* and *lactobacillus* are reduced (Zhou and Zhi, 2016). The change of bacteria of composition in the intestinal tract leads to changes in metabolites (Wishart et al., 2018). Short-chain fatty acids (SCFAs), mainly consisting of acetic acid, propionic acid, and butyric acid, are the main bacterial metabolites (Dabek-Drobny et al., 2022). SCFAs can improve the integrity of the intestinal barrier and maintain intestinal homeostasis (Silva et al., 2018; Lee et al., 2022). The change of SCFAs may play an important role in the pathogenesis of UC.

Modern pharmacological studies have shown that oligosaccharides from plants have a variety of biological functions, including hypoglycemic, antiviral, hypolipidemia, antitumor, and antioxidant (Rastall, 2010; Niu et al., 2021). Fructo-oligosaccharides have a low calorific value, help intestinal absorption of ions, reduce lipid and cholesterol levels, and stimulate the proliferation of *Bifidobacteria* (Bali et al., 2015). Konjac oligosaccharide can decrease the levels of malondialdehyde, inducible nitric oxide synthase, TNF-α, and IL-1β in colonic tissues of rats with colitis, and has significant anti-inflammatory effects (Liu et al., 2016). Therefore, many oligosaccharides are attracting more and more attention as prebiotics functional food ingredients.

Locust bean gum (LBG), can be isolated from the seeds of *Ceratonia siliqua*, commonly found in Mediterranean regions. It is composed of a β-1, 4-linked D-mannose framework and an α-1, 6-linked D-galactose side chain (Barak and Mudgil, 2014). The ratio of mannose to galactose is 4:1 (Tamaki et al., 2010). LBG is added to infant milk powder as a thickener and is commonly used to treat gastroesophageal reflux disease in infants (Alberto Gonzalez-Bermudez et al., 2015; Tounian et al., 2020). Several studies have demonstrated that LBG enzyme hydrolysate can increase the content of beneficial bacteria, inhibit the proliferation of harmful bacteria, and maintain the balance of microecology (Srivastava et al., 2017; Xie et al., 2020; Song et al., 2021). Therefore, we hypothesized that oligosaccharides from LBG could improve dextran sodium sulfate (DSS)-induced colitis by modulating the composition of the intestinal flora. However, enzymatic hydrolysis is limited due to its low efficiency and high cost.

Taken together, in the present study, we attempted to characterize the structural characteristics of the novel hydrolysate from locust bean gum hydrolysate (LBGH) by trifluoroacetic acid (TFA) and to investigate its therapeutic effect in DSS-induced colitis mice. In this study, LBGH structure was determined by thin-layer chromatography (TLC) and high performance liquid chromatography (HPLC) and electrospray ionization (ESI) mass spectrometry (MS). Subsequently, we used the DSS-induced colitis model to evaluate changes in body weight and DAI scores, as well as to evaluate histological morphology, the secretion of cytokines, and the expression of tight junction protein genes and NF-κB P65 protein, the levels of SCFAs, and the composition of intestinal microbiota in C57BL/6 mice before and after LBGH treatment.

## Materials and methods

### Degradable and purification of oligosaccharide

Locust bean gum was purchased from Henan Wanbang Industry Co., Ltd. (China). LBG (5 mg) was hydrolyzed in 100 ml of 0.3 M TFA at 80°C for 2 h (Mao et al., 2021). At the end of the reaction, the precipitate was removed by centrifugation (3,000 *g*, 10 min), The supernatant was dried to solidify in a vacuum oven (XMTD-8222, Jinghong, China) at 15 kPa and 60°C. The solid sample was dissolved by adding 10 ml of methanol and dried again under a vacuum. The above process was repeated 3–5 times to remove TFA. The solid sample obtained was dissolved in 10 ml of distilled water and pre-frozen at −80°C for 12 h, and then freeze-dried in a lyophilizer (Gizs-1, Kaizheng, China) at −30°C and 4 Pa vacuum level for 48 h. Finally, the LBGH was obtained and stored at 4°C until use.

### Chemical and structural characterization of LBGH

The LBGH was analyzed by TLC (Dhalwal et al., 2008). LBGH solution (20 mg/ml) were loaded onto a 50 mm × 100 mm silica gel plate (GF 254, Haiyang, China) and unfolded at room temperature with a solvent system consisting of n-butanol: acetic acid: water (2:2:1, v/v/v). After unfolding, the plate was passed through a color developer (2 ml aniline, 2 g diphenylamine, 10 ml 85% phosphoric acid, and 1 ml hydrochloric acid, dissolved in 100 ml acetone) and heated at 100°C, until the bands were visible.

Structural analysis was performed by HPLC-ESI/MS (Liu et al., 2005) with 1-phenyl-3-methyl-5-pyrazolone (PMP) derivatization (Palanisamy et al., 2017). The LBGH solution was prepared by dissolving 5 mg LBGH into 5 ml distilled water. Then, the 100 μl solution was mixed with 0.3 M NaOH (100 μl) and 0.5 M methanol solution (100 μl) of PMP. The mixture was allowed to react for 1 h at 70°C, then cooled to room temperature, and neutralized with 100 μl 0.3 M hydrochloric acid (HCl). Then, chloroform (700 μl) was added and the mixture was shaken. The chloroform layer was discarded and the extraction process was repeated three times until the chloroform was colorless. The aqueous phase liquid was collected over a 0.22 μm filter membrane (Millex-GP, Merck, Germany) and used for analysis. A Waters Alliance 2487 liquid chromatograph-mass spectrometer (Waters Corporation, United States) equipped with an Agilent 5 HC-C18 (2) column (250 mm × 4.6 mm × 5 μm) and an electrospray detector (ESI) was used. Typical operating conditions were as follows: electrospray voltage was 4 kV; capillary temperature was 350°C; capillary voltage was 48 V; ion lens voltage was 250 V, and the spectra were acquired in the positive mode.

### Animal experiments

**Figure 1:**
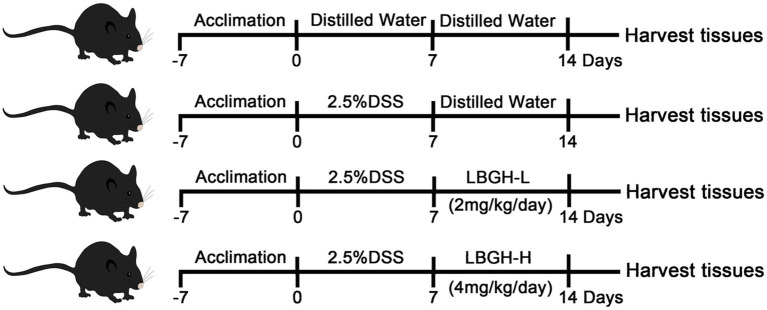
Diagram illustrating the experimental design employed in this study. The concentration of LBGH-L: 2 mg/kg/day; The concentration of LBGH-H: 4 mg/kg/day.

Twenty 7-week-old specific pathogen-free (SPF) male C57BL/6J mice were supplied by Charles River Laboratory Animal Technology (Beijing, China) and housed under autoregulated temperature (25 ± 3°C) and humidity (50 ± 5%) in a 12 h light/dark cycle room. After 1 week of adaptive feeding, 20 mice were divided randomly into four groups named NOR, DSS, LBGH-L, and LBGH-H, respectively. The DSS and LBGH-L, LBGH-H groups were supplied with 2.5% (w/v) dextran sulfate sodium (DSS, 36,000–50,000 Da) in the drinking water for 1 week, while the NOR group drank water freely. The next week, both NOR and DSS groups were given distilled water by gavage, while LBGH-L and LBGH-H were given LBGH aqueous solution with 2 and 4 g/kg/day by gavage, respectively (Figure 1; Mahler et al., 1998). The body weight, food intake, stool characteristics, and bloody feces were measured daily. The disease activity index (DAI) was used to monitor the mice status during the experiment. At the end of the experiment, the mice were sacrificed, blood was collected and centrifuged at 80 *g* for 10 min, and then the serum was obtained. The contents of the cecum and colon, colon tissues, and internal organs, such as the heart, liver, spleen, lungs, stomach, and kidneys were also collected from all animals, and stored at −80°C for further study.

### Histological analysis

Distal colon tissues were fixed in an optimal cutting temperature compound (OCT) embedding medium (Tissue-Tek), frozen at −20°C for 12 h and then cut into 10 μm sections using a frozen sectioning machine (CM1950, Leica, Nussloch, Germany) and stained with hematoxylin and eosin (H&E). In brief, the staining was carried out by the following steps: first, the sections were soaked in 4% formaldehyde fixative for 5 min, in distilled water for 5 min, then sequentially stained with hematoxylin solution for 2 min, soaked in differentiation solution for 5 s, in revertant blue solution for 30 s, stained with eosin solution for 30 s, and rinsed with distilled water for 30 s. The obtained sections were then sequentially dehydrated in graded alcohol (75, 85, 95, and 100%) for 2 s and soaked in xylene for 1 min. Finally, the sections were covered with coverslips and sealed with rhamsan gum. These reagents were purchased from Solarbio Science & Technology Co., Ltd. (Beijing, China). The sections were observed under an upright microscope (Eclipse E200, Nikon, Japan) and photographed using ImageView (Pooher, Shanghai, China). The histologic scores were evaluated as described previously (Guo et al., 2021), a score of 0–4 was given depending on the number of lesions in the colon and their severity.

### Measurement of cytokines

Colonic tissue (100 mg) was mixed with 500 μl pre-cooled RIPA lysis buffer (Beyotime Biotechnology, China) and later ground into a homogenate by a tissue grinder (Wonbio-48P, Shanghai Onebio Biotech Co., Ltd., China). The homogenate was lysed on ice for 20 min and then centrifuged at 14,000 *g* for 5 min at 4°C. The supernatant was collected and stored at −80°C. The protein concentration of the supernatant was determined using a bicinchoninic acid (BCA) protein assay kit (Beyotime Biotechnology, China). The expression levels of cytokines IL-1β, IL-6, and TNF-α in the serum and the colon tissue of mice were analyzed by ELISA kits (Dakewe Biotechnology, Beijing, China) according to the manufacturer’s protocols. For tissues, cytokine levels were determined by dividing the cytokine results (pg/ml) by the measured tissue protein concentration (mg/ml).

### Assessment of tight junction-related gene expression

Total RNA from colon tissues was extracted using TRIzol reagent (Sigma-Aldrich, United States). The purity and concentration of the isolated RNA were determined using NanoDrop 1000 (NanoDrop Technologies; Thermo Fisher Scientific, Inc.). Then, RNA was reverse-transcribed using ABScript II RT Mix for qPCR with gDNA Remover (ABclonal RK20403, China). Real-time quantitative PCR amplification and detection were performed on 2X Universal SYBR Green Fast qPCR Mix (ABclonal RK21203, China) with the specific primers. To normalize mRNA expression, we measured the expression of the glycerol-3-phosphate dehydrogenase (GAPDH). The cDNAs were subjected to qPCR using the following primers (purchased from Sangon Biotech Co., Ltd.): ZO-1 forward, 5′-GCGAACAGAAGGAGCGAGAAGAG-3′ and reverse, 5′-GCTTTGCGGGCTGACTGGAG-3′; Occludin forward, 5′-TGGCTATGGAGGCGGCTATGG-3′ and reverse, 5′-AAGGAAGCGATGAAGCAGAAGGC′; and GAPDH forward, 5′-TGTGTCCGTCGTGGATCTGA-3′ and reverse, 5′-TTGCTGTTGAAGTCGCAGGAG-3′. The following thermocycling conditions were used for qPCR: 95°C for 30 s; 40 cycles at 58°C for 30 s and at 72°C for 30 s; and a final cycle at 72°C for 5 min. The relative expression levels of tight junction-related genes were measured by the 2^−ΔΔCT^ method.

### Assessment of NF-κB p65 protein expression

The frozen colon tissues (100 mg) were added to 500 μl pre-cooled RIPA lysis buffer (Beyotime Biotechnology, China), and then ground thoroughly to obtain mouse colon homogenate. All the homogenate was lysed on ice for 20 min and then centrifuged at 14,000 *g* for 5 min at 4°C. The supernatant was collected and stored at −80°C. The protein concentration of the supernatant was determined using a BCA protein assay kit (Beyotime Biotechnology, China). 15 μg proteins were separated by electrophoresis 12% SDS-PAGE gel and transferred onto a polyvinylidene fluoride (PVDF) membrane. The membrane was blocked with 5% skim milk for 1 h at room temperature and washed with Tris-Buffered Saline Tween-20 (TBST) three times. The PVDF membrane was incubated overnight at 4°C with the primary antibody including β-actin (1:5,000, ABclonal), NF-κB p65 (1:1500, ABclonal). After washed with TBST three times, and incubated with horseradish peroxidase (HRP)-conjugated anti-rabbit immunoglobulin IgG (1:2, 000, ABclonal) for 1 h at 4°C, and washed with TBST three times. The enhanced chemiluminescence (ECL) reagent was used for signal detection (GE Amersham Imager 600, United States). The gray value was analyzed with Image J software (US National Institutes of Health, Bethesda). The relative protein expression levels were normalized by the results of β-actin.

### Quantitative analysis of SCFAs

The short-chain fatty acids (SCFAs) concentration in colon contents was determined by Gas Chromatography-Mass Spectrometer (GC–MS, Agilent J&W DB-FFAP), an Agilent VF-WAXms 30 m × 0.25 mm × 0.25 μm column was used. The appropriate amount of colon contents was mixed with five times the volume of water by vortexing and the mixture was centrifuged at 13,000 *g* for 10 min at 4°C. The resulting supernatant of 200 μl was mixed with 20 μl of 2-ethylbutyric acid (internal standard), 500 μl HCl (37%), and 2 ml diethyl ether and vortexed for 3 min. The supernatant was transferred to a sterile centrifuge tube, anhydrous sodium sulfate (Na_2_SO_4_) was added and vortexed for 2 min. After standing for 5 min, the supernatant was taken and filtered through a 0.22 μm membrane. Typical operating conditions were as follows: the temperatures of the inlet, ion source, and transfer line were set to 240°C. The column temperature was set to an initial temperature of 100°C for 0.5 min, held for 2 min from 8°C/min to 200°C, and held for 1 min from 10°C/min to 240°C. The energy of electron ionization was set to 70 eV.

### Gut microbiota analysis

Total genome DNA from cecum contents was extracted using the CTAB method. DNA concentration and purity were monitored on 1% agarose gels. According to the concentration, DNA was diluted to 1 ng/μl using sterile water. The V3–V4 region of the 16S rRNA gene was amplified by PCR with barcode specific primer (338F: 5′-ACTCCTACGGGAGGCAGCAG-3′ and 806R: 5′-GGACTACHVGGGTWTCTAAT-3′) using Phusion® High-Fidelity PCR Master Mix (New England Biolabs). PCR products were purified with Qiagen Gel Extraction Kit (Qiagen, Germany) and DNA libraries were constructed using TruSeq® DNA PCR-Free Sample Preparation Kit (Illumina, San Diego, United States). The library quality was assessed on the Qubit@ 2.0 Fluorometer (Thermo Scientific). At last, the library was sequenced on an Illumina NovaSeq platform and 250 bp paired-end reads were generated. Raw data were analyzed using the QIIME2 platform. Sequencing service and data analysis service were provided by Wekemo Tech Group Co., Ltd. (Shenzhen China).

### Statistical analysis

All the experimental data (*n* = 5 per group) were shown as the mean ± SD. SPSS (Version 11.5, IBM, United States) was used to perform all the statistical analyses. GraphPad Prism 7 software (GraphPad Software, San Diego, CA, United States) was used for graphical processing. Using Tukey’s *t*-test for comparison or one-way ANOVA for multiple comparisons to measure the value of *p* of the difference between groups. There is a significant difference when *p* < 0.05.

## Results

### Chemical structure of LBGH

**Figure 2:**
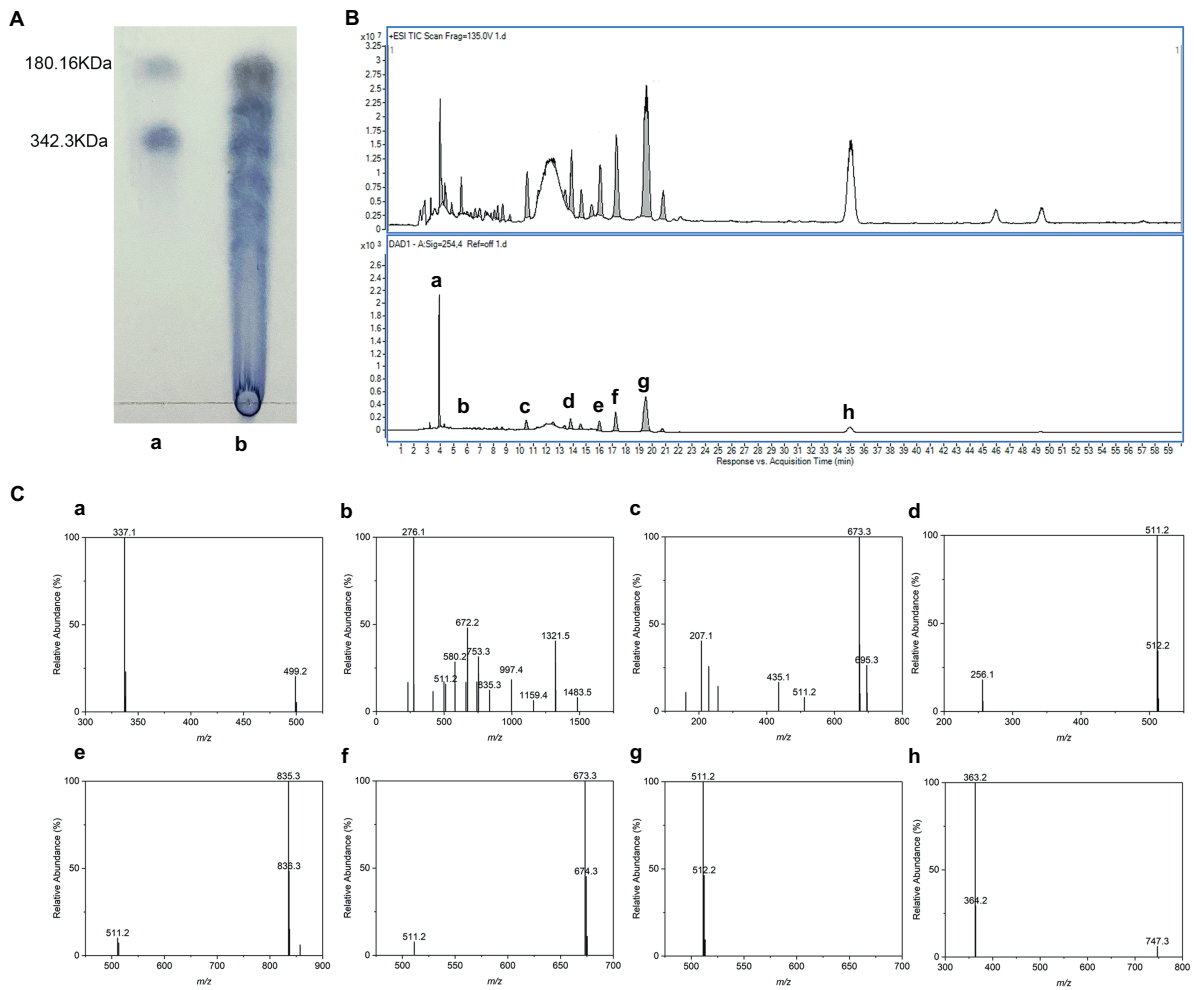
Chemical structure of locust bean gum hydrolysate (LBGH). **(A)** Thin-layer chromatography (TLC) analysis of LBGH; **(B)** High performance liquid chromatography (HPLC)-UV chromatogram of LBGH; **(C)** Positive mode electrospray ionization (ESI)-mass spectrometry (MS) analysis of partially collected components **(a–h)** by HPLC **(B)**. **(a)** m/z 337: [M2 + 2PMP - H2O + H] + − Gal–PMP; m/z 499: [M2 + PMP-H2O + H] + -PMP; **(b)** m/z 511, 835, 997, 1,159, 1,321, and 1,483: [M1,3–7 + 2PMP-H2O + H]+; m/z 672: [M2 + 2PMP-H2O]+; **(c)** m/z 511, 673: [M1-2 + 2PMP-H2O + H]+; m/z 695: [M + 2PMP-H2O + Na]+; **(d)** m/z 511: [M1 + 2PMP-H2O + H]+; **(e)** m/z 511, 835: [M1,3 + 2PMP-H2O + H]+; **(f)** m/z 511, 673: [M1,2 + 2PMP-H2O + H]+; **(g)** m/z 511: [M1 + 2PMP-H2O + H]+; and **(h)** m/z 363: The dimer is present as a sodium adduct and is oxidized to aldehyde or ketone.

Thin-layer chromatography (TLC) results showed that LBG was degraded to oligosaccharides and monosaccharides with a lower DP (Figure 2A). To figure out the compositional information including the DP of individual oligosaccharides and type of constituent monosaccharides, LBGH was analyzed by HPLC-ESI/MS after PMP derivatization. There were eight main peaks in the HPLC chromatograms of LBGH (Figure 2B). Their structure was illustrated further by ESI-MS analysis. Figure 2C shows the mass spectra of the ion peaks obtained from the analysis of each component in positive ion mode.

All [M + 2PMP - H_2_O] ions identified in this spectrum and their relative abundance are displayed in Figure 2C. The peaks m/z 499 and 337 in Figure 2C–a correspond to the [M_2_ + 2PMP - H_2_O + H]^+^ consecutive loss of a galactosyl residue and a PMP group. Figures 2C-b–g show the ions observed at m/z 511, 672, 835, 997, 1,159, 1,321, and 1,483 can be summarized as a series of derivatives from monosaccharide to heptasaccharide with PMP [M_1-7_ + 2PMP−H_2_O + H]^+^. Corresponding sodium addition is observed for the ion at m/z 695 ([M_2_ + 2PMP−H_2_O + Na]^+^) in Figure 2C-c. The dimer (Figure 2C-h) is present as a sodium adduct and is oxidized to aldehyde or ketone (m/z 363; Ponzini et al., 2019). In summary, we suggested that the acid digestion products of LBG consisted of a mixture of monosaccharides and oligosaccharides with DP 2–7, of which monosaccharides and disaccharides accounted for the majority. The results of the ESI/MS were consistent with those of the TLC experiments.

### LBGH ameliorated colitis symptoms in DSS-treated mice

**Figure 3:**
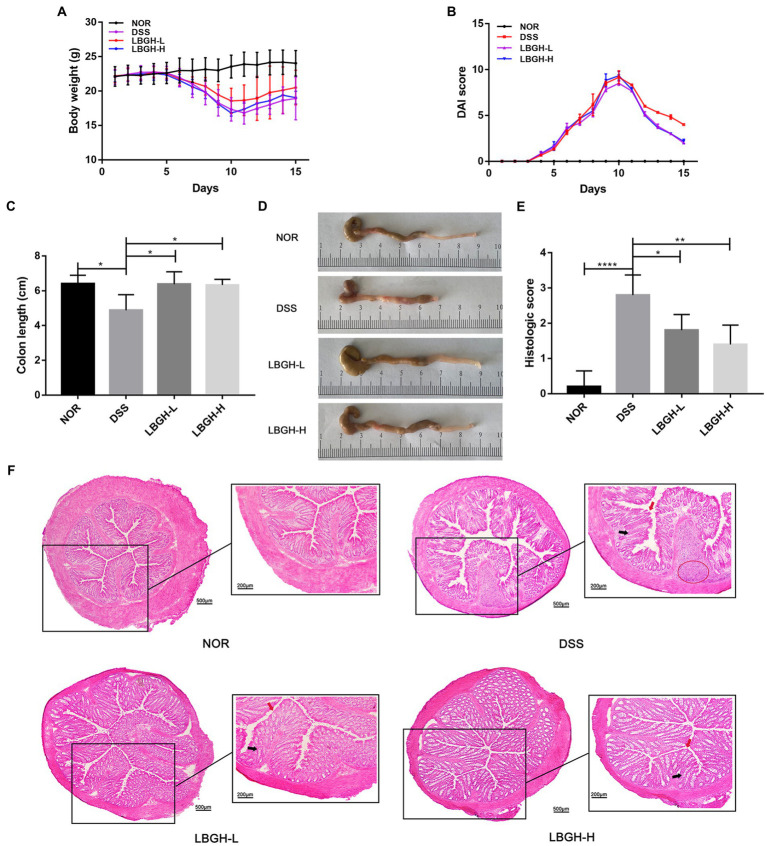
LBGH ameliorates dextran sulfate sodium (DSS)-induced colitis in mice. **(A)** Daily body weight changes following DSS treatment. **(B)** Disease activity index changes following DSS treatment. **(C)** The lengths of the colon from each group. **(D)** The representative picture of the colon. **(E)** Histologic score. **(F)** Representative hematoxylin and eosin (H&E)-stained distal colon sections. The highlights are the inflammatory infiltrate (red circle), the villi surface (red arrow), and the crypt (black arrow). *p < 0.05, **p < 0.01, and ****p < 0.001 compared with the DSS group. (*n* = 5 per group).

The experimental design of the mouse feeding test is shown in Figure 1. Changes in body weight, DAI and colonic length in mice are shown in Figures 3A,B. Over the experiment, the weight of the NOR mice showed a gradual upward trend. In contrast, on the 6th day of the experiment, mice began to lose weight due to the intervention of DSS (Figure 3A). On the 11th day, the mice in DSS group recovered to their normal diet and the weight gained slowly, while the LBGH groups began to gain weight on the 10th day.

The DAI in the DSS and LBGH groups increased continuously from day 3 to day 10 and began to decrease after day 11 (Figure 3B). Treatment with LBGH improved weight in mice and ameliorated diarrhea and bloody stools.

In DSS-induced colitis, the length of colon can be used as an indicator to reflect the severity of inflammation. The colon length in the DSS group was significantly shorter than that in the NOR group (*p* < 0.05). However, LBGH alleviated this situation, with no significant difference in colonic length compared to the NOR group (Figures 3C,D). These results showed that LBGH improved the symptoms of colitis, but there was no significant difference in the treatment effect between high and low dose.

### LBGH reduced the histological injury of colon

Histologically, the intervention of the DSS significantly distorted the structure of the crypt, causing irregularities in the surface of the villi and marked inflammatory cell infiltration (Figure 3F). However, LBGH reduced the infiltration of inflammatory cells and the damage to the crypt. This was confirmed by the histological score (Figure 3E).

### LBGH inhibited inflammatory cytokine secretion

**Figure 4:**
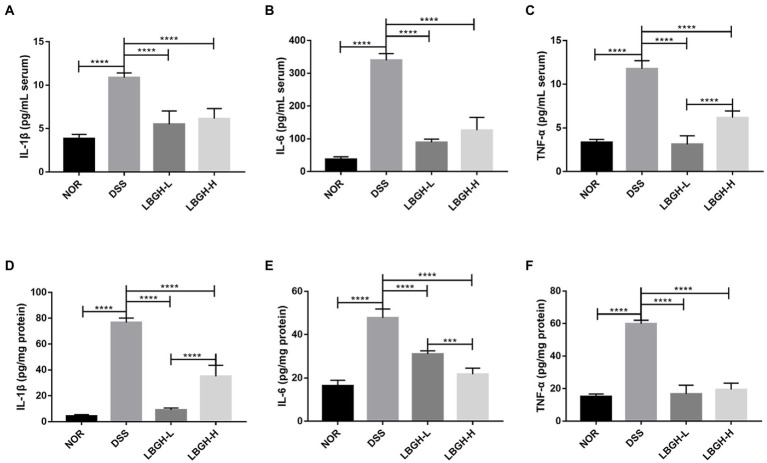
LBGH inhibits inflammatory cytokine secretion. **(A–C)** Inflammatory cytokines including IL-1β, IL-6, and TNF-α in serum of mice. **(D–F)** Inflammatory cytokines including IL-1β, IL-6, and TNF-α in the colon tissue of mice. ***p < 0.001 and ****p < 0.0001 compared with the DSS group. (*n* = 5 per group).

To further assess the effect of LBGH on DSS-induced colitis in mice, we measured the levels of pro-inflammatory cytokines including IL-1β, IL-6, and TNF-α in serum as well as in colonic tissue through ELISA kits. DSS intervention significantly increased the levels of pro-inflammatory cytokines compared with the NOR group both in serum and colonic tissue (*p* < 0.0001). The pro-inflammatory cytokines decreased significantly in mice after being treated with LBGH (Figures 4A–F; *p* < 0.0001). These results demonstrated that LBGH attenuated the inflammation of DSS-induced colitis in mice.

### LBGH regulates the expression of tight junction-related proteins

**Figure 5:**
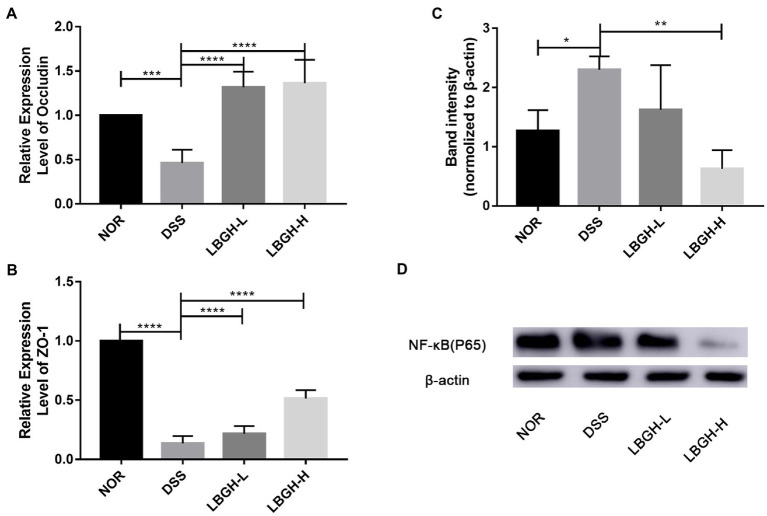
LBGH regulates the expression of tight junction-related proteins and LBGH activates NF-κB pathway. **(A)** The gene transcription levels of Occludin. **(B)** The gene transcription levels of ZO-1. **(C,D)** The level of NF-κB (p65) protein is normalized relative to that of β-actin and the phosphorylation levels are quantified by Image J. *p < 0.05, **p < 0.01, ***p < 0.001, and ****p < 0.0001 compared with the DSS group. (n = 5 per group).

To investigate the effect of LBGH on intestinal tight junctions, gene expression levels of ZO-1 and Occludin were measured in colonic tissue. The expression of ZO-1 and Occludin protein was significantly decreased in the DSS group compared to the NOR group (*p* < 0.001; Figures 5A,B). While the use of LBGH (low dose and high dose) significantly reversed the levels of ZO-1 and Occludin (*p* < 0.0001).

### LBGH suppressed the activation of NF-κB (p65)

We further analyzed the inflammatory signaling pathway by Western blot and the intensity of the bands was quantified by Image J. Compared to the NOR group, NF-κB (p65) was activated in mice treated by DSS, whereas it was significantly inhibited by LBGH (Figures 5C,D). In particular, the expression of NF-κB (p65) protein was significantly decreased in the LBGH-H group compared to the DSS group (*p* < 0.01). The available data suggested that LBGH could alleviate inflammation which encouraged us to find the underlying mechanism.

### LBGH promoted the production of SCFAs

**Figure 6:**
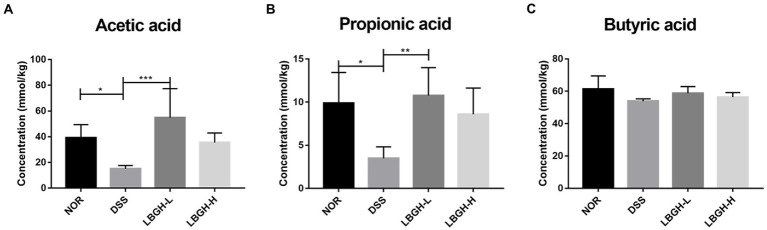
LBGH promotes the production of short-chain fatty acids (SCFAs). **(A)** Concentrations of fecal acetic acid. **(B)** Concentrations of fecal propionic acid. **(C)** Concentrations of fecal butyric acid. *p < 0.05, **p < 0.01, and ***p < 0.001 compared with the DSS group. (*n* = 5 per group).

Short-chain fatty acids are products of intestinal bacterial metabolism and include mainly acetic acid, propionic acid, and butyric acid (Peng et al., 2013; Venegas et al., 2019). The amount of SCFAs in the colon of each group was determined by GC–MS (Figure 6). The content of acetic acid and propionic acid was significantly lower in the DSS group compared to the NOR group (*p* < 0.05). In addition, after treatment with the LBGH, the contents of acetic acid and propionic acid were significantly increased compared with those of the DSS group (*p* < 0.05). Our results indicated that LBGH promoted the levels of SCFAs, which was beneficial for alleviating intestinal inflammation.

### LBGH regulated gut microbiota

**Figure 7:**
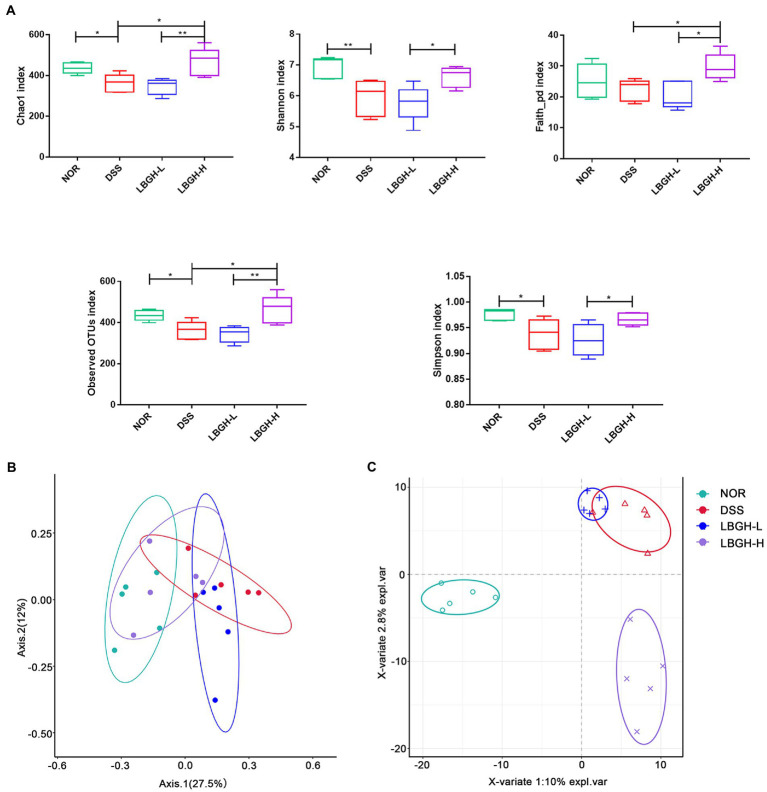
LBGH regulates gut microbiota diversity. **(A)** The alpha diversity (Chao1, Shannon, Faith_pd, Observed OTUs, and Simpson species indexes) in different groups. **(B)** The beta diversity by principal coordinate analysis (PCoA) plot of Bray Curtis distances and Partial Least Squares Discriminant Analysis (PLS-DA). Significant differences are indicated as *p < 0.05 and **p < 0.01. (*n* = 5 per group).

We investigated whether LBGH altered the composition of the gut microbiota of mice with DSS-induced colitis by 16S rDNA high-throughput sequencing. The Chao1, Shannon, Faith_pd, Simpson, and Observed OTUs indexes were used to estimate the alpha diversity among each group. The results showed that the intervention of LBGH-H significantly improved the alpha diversity of mice with colitis (Figure 7A). Principal coordinate analysis (PCoA) using Bray Curtis distances and Partial Least Squares Discriminant Analysis (PLS-DA) were performed to assess beta diversity. As shown in Figures 7B,C, there was a distinct separation on the beta diversity of gut microbial communities between any two groups. These results displayed that LBGH significantly altered the alpha and beta diversity.

**Figure 8:**
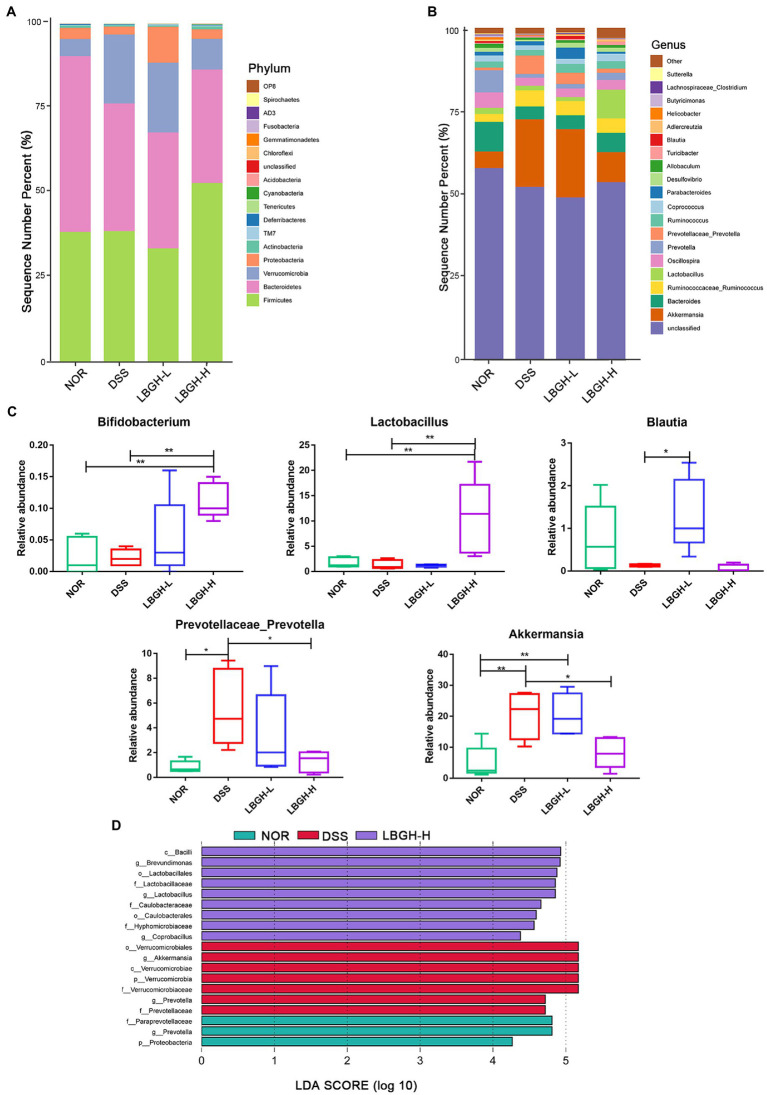
LBGH regulates gut microbiota in DSS-induced colitis mice. **(A,B)** Gut microbiota composition at phylum level and genus level. **(C)** Changes in the composition of the intestinal microbiota at the genus level. Significant differences are indicated as *p < 0.05 and **p < 0.01. **(D)** Histogram of LDA value distribution of the differential microbial community (LDA score threshold of >4). (*n* = 5 per group).

To determine the species composition of the gut microbial community, we analyzed differences in the gut microbiota at the level of phylum and genus. At the phylum level, 17 phyla are identified in all samples as shown in Figure 8A. The DSS supplementation significantly increased the *Verrucomicrobia* but decreased the *Bacteroidetes* by comparison with the NOR group. Interestingly, high dose of LBGH effectively reduced the level of *Verrucomicrobia* in contrast to DSS-induced colitis mice, and increased the *Firmicutes*. Figures 8B,C showed the gut microbiota compositions at the genus level in each group of mice. The high dose of LBGH significantly increased the relative abundance of *Bifidobacterium* and *Lactobacillus* compared to the DSS and NOR groups (*p* < 0.01). The relative abundance of *Prevotellaceae_Prevotella*, *Akkermansia* in the DSS group was significantly increased compared to the NOR group, whereas high dose of LBGH treatment reduced the relative abundance to a level close to that seen in the NOR group. The low dose of LBGH significantly increased the relative abundance of *Blautia* compared to the DSS group (*p* < 0.05). Thus, these results suggested that LBGH alleviated gut microbiota dysbiosis and profoundly modulated gut microbiota composition in mice with DSS-induced colitis.

The linear discriminant analysis (LDA) effect size (LEfSe) was used to identify statistically significant biomarkers and reveal the dominant microorganisms in each group (Figure 8D). We found that nine bacterial genera including *Lactobacillus* were enriched in the LBGH-H group, and seven bacterial genera including *Akkermansia* were enriched in the DSS only group. *Prevotella* was the dominant taxa in the NOR group.

**Figure 9:**
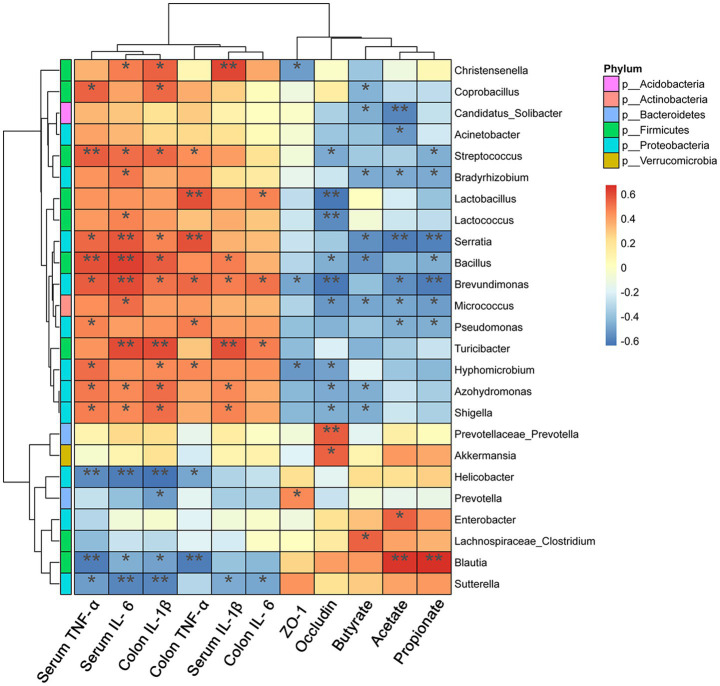
Heat map representation of Spearman’s correlation coefficient between bacterial taxa and colitis-related index. The red color denotes a positive correlation, while the blue color denotes a negative correlation. Significant differences are indicated as *p < 0.05, **p < 0.01, and ***p < 0.001 (*n* = 5 per group).

Correlations between significantly altered taxa in the gut microbiome and colitis-related indexes were investigated using Spearman correlation analysis. Twelve different bacterial genera of *Firmicutes* and *Proteobacteria* showed a significant positive correlation (*p* < 0.05) with the concentration of pro-inflammatory mediators in serum and colon (Figure 9). *Akkermansia* and *Prevotellaceae-Prevotella* were positively correlated with the Occludin level (*p* < 0.05). Moreover, the SCFAs level showed a significantly positive correlation with the abundance of *Blautia* (*p* < 0.01), but it presented a significantly negative correlation with *Proteobacteria* (*p* < 0.05).

## Discussion

In recent years, the degraded galactomannan has attracted great interest because of its various biological activities. It has been demonstrated that incomplete degradation products of galactomannans can improve antioxidant function by increasing antioxidant content, and modulate immune responses by increasing the secretion of immunoglobulin and cytokines (Tao et al., 2021). Galactomannan is the main component of LBG (Williams and Phillips, 2009). In this study, LBG was degraded by TFA to obtain LBGH, which was composed of monosaccharides and oligosaccharides with DP of 2–7. Therefore, based on the structural characteristics and relevant studies, we further explored the biological activities of the LBGH in mice. Our results demonstrated that LBGH attenuated colonic inflammation and modulated intestinal flora in mice with DSS-induced colitis.

Cytokines play a key role in the intestinal immune response (Moldoveanu et al., 2015; Marafini et al., 2019). Overproduction of pro-inflammatory cytokines, such as TNF-α, IL-1β, and IL-6, is the typical feature of the DSS-induced colitis mouse model (Randhawa et al., 2014; Wang et al., 2018). In the present study, LBGH treatment significantly inhibited the overproduction of pro-inflammatory cytokines in DSS-induced colitis mice. Compared with the NOR group, the serum levels of TNF-α, IL-6, and IL-1β were increased by 71.58, 89.11, and 64.67%, respectively, in the mice of the DSS group. And after LBGH intervention, the levels of TNF-α, IL-6, and IL-1β were reduced by 73.51, 73.78, and 49.47%, respectively, in the low-dose LBGH group, compared to the DSS group. We also found that the levels of TNF-α, IL-6, and IL-1β in mouse colonic tissue showed the same trend. These data indicated that serious inflammatory response had happened in mice colon and tissue damage might come out simultaneously. Therefore, administration of the LBGH at different doses could ameliorate the inflammation by inhibiting the production of pro-inflammatory cytokines in the DSS group.

The expression of the transcription factor NF-κB is often accompanied by increased production of interleukin (IL-1β and IL-6) and TNF-α (Neurath et al., 1998). We also discovered that DSS activated the signaling pathway NF-κB and increased IL-1β, IL-6, and TNF-α in DSS-induced colitis mice. The intervention of LBGH inhibited the signaling pathway NF-κB and reversed the levels of the interleukin and TNF-α. Therefore, we speculated that the regulation of IL-1β, IL-6, and TNF-α by LBGH was associated with the NF-κB signaling pathway.

The intestinal barrier is an important part of maintaining intestinal health (Johansson et al., 2013). Tight junction complex proteins including ZO-1(Kuo et al., 2021), and Occludin (Cummins, 2012) are important components of the intestinal mechanical barrier. In the present study, the mRNA expression of Occludin and ZO-1 proteins in DSS-induced colitis mice was reduced by 53.73 and 86.46%, respectively. However, high-dose LBGH treatment increased the relative expression levels of both by 66.03 and 73.65%, and they showed an increasing trend in a dose-dependent manner in the oligosaccharide-treated group. In addition, the integrity of the intestinal barrier was considerably better in LBGH group than in the DSS group according to H&E staining of colonic tissue.

There is a general and persistent hypothesis that a large number of lactic acid bacteria species form a stable and important part of the human gut and that damage to the intestinal barrier can be prevented by regulating the distribution and expression of tight junction proteins (Siew-Wai et al., 2010; Vemuri et al., 2017). In our study, it is worth noting that LBGH had a positive effect on increasing the abundance of *Lactobacillus* and *Bifidobacterium* (*p* < 0.01). Both the abundance of *Lactobacillus* and the levels of ZO-1 and Occludin were increased by LBGH in DSS-induced colitis mice, which meant LBGH was beneficial for the intestinal tight junctions.

The gut microbiota can use indigestible carbohydrates to produce SCFAs, which are the main energy source of the colonic epithelium (Arpaia et al., 2013). It has also been reported that SCFAs are closely associated with inflammation. They protect the integrity of the intestinal epithelium and promote the intestinal immune response, thereby protecting the intestinal wall and reducing the incidence of intestinal inflammation (Liu et al., 2021). It has been demonstrated that SCFAs can effectively inhibit the inflammatory response of Caco-2 cells and maintain the tight junctions of intestinal mucosal epithelial cells (Xi et al., 2022). Li et al. reported that supplementation of the basal diet with SCFAs could protect the zebrafish against pathogenic bacteria, modulate the gut microbiota, and enhance the immune response in the host (Li et al., 2022). In the DSS group, the levels of acetic acid, propionic acid, and butyric acid were 61.38, 64.82, and 12.10% lower than in the NOR group. However, low dose of LBGH increased its levels by 3.62, 3.09, and 1.09 times, respectively. We also found that low dose of LBGH increased the abundance of *Blautia* by 7.08 times. The fermentation products of *Blautia* such as acetic acid, propionic acid, and butyric acid have an excellent anti-inflammatory effect in mice with colitis (Guo and Li, 2019). LBGH had increased the content of acetic acid, propionic acid, and butyric acid, which is consistent with the increased abundance of some SCFAs-producing bacteria, like *Blautia* in LBGH groups.

The relative abundance of *Verrucomicrobia* was significantly increased in the DSS group in this study, mainly due to the prevalence of *Akkermansia-Muciniphila*. *Akkermansia-muciniphila* is a commensal bacterium of the mucous layer that degrades mucin as its sole source of energy (Zhai et al., 2019). Wu et al. found that the relative abundance of *Akkermansia* is positively correlated with the production of SCFAs (Wu et al., 2021). It has been shown that cranberry extract improves insulin resistance in diet-induced obese mice by increasing the abundance of *Akkermansia*, which reduces intestinal permeability and LPS leakage (Anhe et al., 2015). These studies all suggested that *Akkermansia* has beneficial effects on intestinal inflammation. Interestingly, Cai et al. reported an abnormal amount of *Akkermansia* in the DSS group, possibly due to DSS-induced lesions in the mucus layer (Cai et al., 2019). In addition, it has been reported that treatment with flaxseed oligosaccharides can inhibit the over-proliferation of *Akkermansia* in DSS-induced colitis mice (Xu et al., 2020). These observations were consistent with the fact that decreased abundance of *Akkermansia* was observed in mice with colitis treated by high dose of LBGH.

Therefore, the beneficial effect observed in this study may be due to that LBGH can increase the number of beneficial bacteria and maintain the balance of the intestinal flora.

## Conclusion

This is a new study focusing on the structure of LBGH and its anti-inflammatory activity *in vivo*. Our results indicate that the LBGH inhibited the production of proinflammatory factors and suppressed the activation of the NF-κB pathway, enhanced the expression of tight junction proteins (Occludin, ZO-1) and the concentration of SCFAs. Furthermore, LBGH modulated the gut microbiota, enriched intestinal microbial diversity, and significantly increased the abundance of *Lactobacillus* and *Bifidobacterium*. In conclusion, LBGH has shown the ability to relieve DSS-induced colitis.

## Data availability statement

The datasets presented in this study can be found in online repositories. The names of the repository/repositories and accession number(s) can be found at: NCBI—PRJNA857152.

## Ethics statement

The animal study was reviewed and approved by all animal experiments complied with the ARRIVE guidelines and were carried out in accordance with the U.K. Animals (Scientific Procedures) Act, 1986 and associated guidelines, EU Directive 2010/63/EU for animal experiments, the National Institutes of Health guide for the care and use of Laboratory Animals (NIH Publications no. 8023, revised 1978), and the Animal Management Rules of the Chinese Ministry of Health (no. 55, 2001). This study was approved by the Animal Experiment Ethics Committee of Qilu University of Technology (Jinan, China).

## Author contributions

KJ and SZ: conceptualization, methodology, resources, and writing—review and editing. KJ and DW: software, formal analysis, investigation, and data curation. KJ, SZ, and DW: validation. KJ: writing—original draft preparation and visualization. SZ, LS, XL, QY, and LZ: supervision. LZ: project administration. LZ, KL, and BL: funding acquisition. All authors contributed to the article and approved the submitted version.

## Funding

This research was funded by Shandong Taishan leading talent project (grant number LJNY202015), the Science Foundation of China (grant number 31501396), Spring Industry Leader Talent Support Plan (grant number 2019042), Yantai Development Zone Science and Technology Leading Talents Project (grant number 2020CXRC4, Lin Zhao, and Orlando Borrás—Hidalgo), National key plan “science and Technology to help the economy” special project (grant number SQ2020YFF0401390), and University, government, industry, research Collaborative innovation Fund project (2020-CXY45).

## Conflict of interest

BL and KL were employed by the company Shangdong Zhuoran Biotechnology Co., Ltd. LZ was employed by the company Shandong Chenzhang Biotechnology Co., Ltd.

The remaining authors declare that the research was conducted in the absence of any commercial or financial relationships that could be construed as a potential conflict of interest.

## Publisher’s note

All claims expressed in this article are solely those of the authors and do not necessarily represent those of their affiliated organizations, or those of the publisher, the editors and the reviewers. Any product that may be evaluated in this article, or claim that may be made by its manufacturer, is not guaranteed or endorsed by the publisher.
